# Characterization of a New Mouse Model for Peripheral T Cell Lymphoma in Humans

**DOI:** 10.1371/journal.pone.0028546

**Published:** 2011-12-05

**Authors:** Niklas Beyersdorf, Sandra Werner, Nelli Wolf, Thomas Herrmann, Thomas Kerkau

**Affiliations:** University of Würzburg, Institute for Virology and Immunobiology, Würzburg, Germany; Centre de Recherche Public de la Santé (CRP-Santé), Luxembourg

## Abstract

Peripheral T cell lymphomas (PTCLs) are associated with a poor prognosis due to often advanced disease at the time of diagnosis and due to a lack of efficient therapeutic options. Therefore, appropriate animal models of PTCL are vital to improve clinical management of this disease. Here, we describe a monoclonal CD8^+^ CD4^−^ αβ T cell receptor Vβ2^+^ CD28^+^ T cell lymphoma line, termed T8-28. T8-28 cells were isolated from an un-manipulated adult BALB/c mouse housed under standard pathogen-free conditions. T8-28 cells induced terminal malignancy upon adoptive transfer into syngeneic BALB/c mice. Despite intracellular expression of the cytotoxic T cell differentiation marker granzyme B, T8-28 cells appeared to be defective with respect to cytotoxic activity as read-out *in vitro.* Among the protocols tested, only addition of interleukin 2 *in vitro* could partially compensate for the *in vivo* micro-milieu in promoting growth of the T8-28 lymphoma cells.

## Introduction

To develop novel therapeutic strategies for peripheral T cell lymphoma (PTCL) appropriate animal models are crucial [1]. While researchers have been isolating, and also wrongly re-isolating [2], transplantable T cell lymphoma lines since the 1940s from mice after chemical tumor induction/promotion [3], only recently the ectopic expression of an inducible T cell kinase (ITK)-spleen tyrosine kinase (SYK) fusion gene has allowed to establish the first mouse model of PTCL [1], [4], [5]. To what extent even these genetically induced neoplasias resemble their human pendants is, of course, unclear. Therefore, isolation and characterization of the T8-28 cell line from an un-manipulated BALB/c mouse will be instrumental in furthering our understanding of lymphomagenesis in mice and will help to develop successful therapies for PTCL in humans.

## Results and Discussion

**Figure 1:**
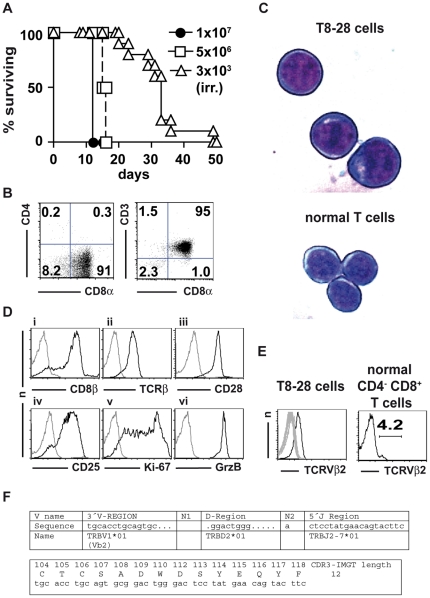
*In vivo* tumorigenicity of and T cell marker expression by T8-28 cells. (A) Survival of unmanipulated BALB/c.OlaHsd mice transplanted with 1×10^7^ (experiment 1; n = 2) or 5×10^6^ (experiment 2; n = 2) T8-28 cells or irradiated BALB/c.OlaHsd mice transplanted with 3×10^3^ T8-28 cells on day 0 (pooled data of two experiments; n = 10). (B) CD4, CD8α and CD3 expression by T8-28 cells. Figures indicate the percentages of cells in the respective quadrants. (C) Cytospins of T8-28 and freshly isolated mouse T cells stained by the Diff-Quik® method. Original magnification was 630-fold. Microscope: LEICA DMIRE2. Camera: LEICA DFC300 FX. Acquisition Software: LEICA IM50 Image Manager. Adobe Photoshop CS3 was used to obtain the close-ups shown. (D) Fluorescence-activated cell sorting analysis of T cell lineage and activation markers as indicated (black histograms). Staining controls (grey histograms): i, v, isotype-matched control mAb; ii,vi, non-isotype-matched control mAb; iii, no primary mAb; iv: identical control staining as in iii. (E) Expression of TCRVβ chains by T8-28 cells and CD4^−^ CD8^+^ T cells of a normal BALB/c mouse as indicated. Grey histograms represent overlays of the 14 remaining TCRVβ chains (Vβ3–Vβ18) detected by the kit used. (F) The genetic setup and primary structure of the TCR CDR3 region was analyzed by polymerase chain reaction DNA sequencing and the IMGT/V-QUEST program. Please note that TRBV1 in the IMGT nomenclature is identical to designation as Vβ2 used throughout this paper and that the *01 allele has originally been found in BALB/c mice which is consistent with the origin of the T8-28 cells. The sequenced Cβ part (not shown) could be identified as Cβ2 as expected for a TCR-chain using the second DJ cluster.

T8-28 cells were initially isolated from an un-manipulated adult male BALB/c.OlaHsd mouse kept under standard housing conditions. The animal was found to be paraparetic and, thus, killed for humane reasons. Upon necropsy, spleen and lymph nodes were grossly enlarged containing 1.0×10^9^ and 1.7×10^9^ cells, respectively. To determine, whether the secondary lymphoid organ cell suspensions of this animal contained transplantable tumor cells, we injected 1×10^7^ or 5×10^6^ splenocytes intravenously into syngeneic BALB/c mice, which induced terminal malignancy in the recipient mice after 12 and 16 days, respectively, (Figure 1A) with splenocyte numbers ranging from 4.5×10^8^ to 1.7×10^9^ cells in post-mortem analyses. Tumorigenicity of the splenocytes we had isolated was further tested under clinically relevant conditions [6] by injecting 3×10^3^ cells into lethally irradiated BALB/c.OlaHsd mice followed by re-constitution of hematopoiesis using T cell-depletetd bone marrow cells isolated from C57BL/6 mice. In this scenario terminal malignancy developed with a median of 33 days (Figure 1A).

The splenocytes we had transplanted consisted by more than 95% of CD8αβ^+^ CD4^−^ CD3^+^ TCR^+^ and CD28^+^ T cells as revealed by fluorescence-activated cell sorting analysis (Figure 1B, D), indicating that we had isolated a T cell lymphoma which we termed T8-28. Moreover, comparing the lymphoma cells with freshly isolated normal mouse T cells by light microscopy showed that they, indeed, had a lymphoblastic morphology (Figure 1C) further corroborating our diagnosis.

The overall expression of lineage and activation markers, including CD25, Ki-67 and granzyme B (Figure 1D and Table S1), suggested that the T8-28 cells largely resembled fully differentiated cytotoxic T cells with an, at most, oligoclonal TCR repertoire comprising only Vβ2^+^ cells (Figure 1E). Polymerase chain reaction DNA sequencing confirmed an in-frame rearrangement of TCR Vβ2 and produced a single complementarity-determining region (CDR) 3 sequence revealing that the T8-28 cells actually constitute a monoclonal population. Moreover, knowledge of the CDR3 sequence used by T8-28 cells will allow to track micro-metastases in future experiments *in vivo*. Therefore, the T8-28 lymphoma is a peripheral T cell lymphoma most likely stemming from a transformed mature CD8^+^ T cell.

**Figure 2:**
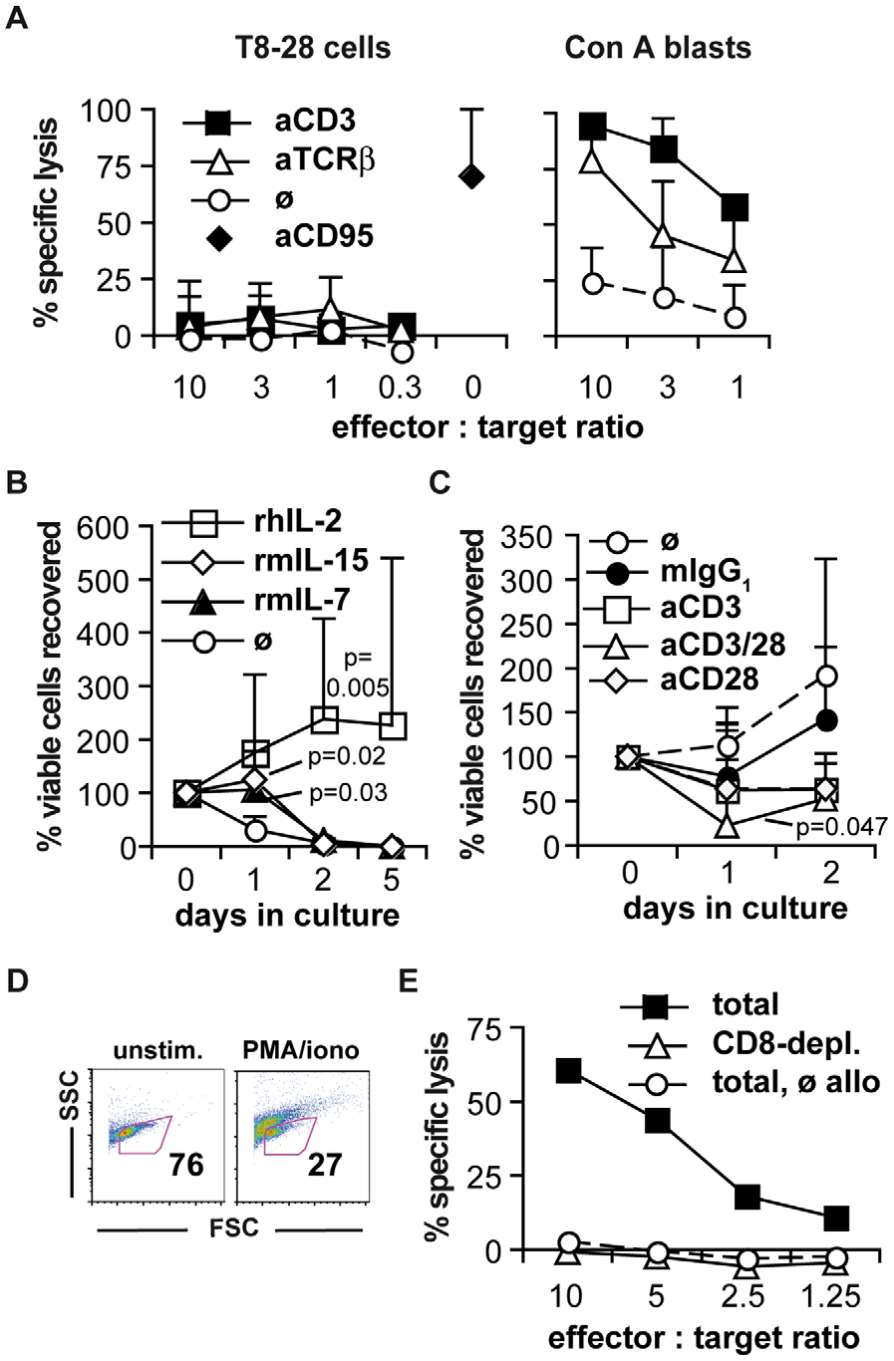
T cell effector functions of T8-28 cells and susceptibility to killing by allogeneic T cells. (A) T8-28 cells (left) or Con A blasts (right) were used as effector cells in re-directed lysis assays against A20J cells in the presence or absence of the indicated antibodies. Means ± SD of three to six independent experiments are shown. (B) Freshly thawed T8-28 cells were cultured either alone or in the presence of 10^−6^ M of the indicated cytokines. Means ± SD of two to five experiments are shown. (C) Cell recovery after culture of T8-28 cells in the presence of 10^−6^ M rhIL-2 and paramagnetic beads coated with anti-CD3 and/or anti-CD28 mAbs as indicated. P-values in (B) and (C) are the results of two-way ANOVA testing against the cells without the specified treatment (‘ø’). (D) Percentage of viable cells as determined by FSC/SSC characteristics among un-stimulated and PMA/ionomycin re-stimulated T8-28 cells. The figures indicate the number of cells within the life gate. The data are representative for more than three experiments with comparable results. (E) T8-28 cells are susceptible to killing by allogeneic CD8^+^ T cells. CD8^+^ cell-depleted allogeneic lymph node cells (triangles) and lymph node cells cultured without allogeneic APCs (circles) were used as controls. Means of duplicates are shown. The experiment was repeated with similar result.

Although T8-28 cells expressed markers of cytotoxic T cells they, however, failed to mediate cytotoxicity in re-directed lysis assays against A20J target cells which were readily killed by syngeneic Con A blasts after TCR cross-linking (Figure 2A). Apart from defective effector functions, de-differentiation of malignant cells, of course, also manifests in uncontrolled growth. Therefore, we analyzed the growth characteristics of T8-28 cells *in vitro*. Culture of T8-28 cells in medium only led to a rapid decline in cell numbers which was stalled for one day by the addition of recombinant mouse interleukin 15 or 7 and partially reverted by the addition of recombinant human interleukin 2 (Figure 2B). So far, long-term *in vitro* culture of the T8-28 cells beyond day five has, however, not been possible.

Combining recombinant human interleukin 2 with stimulation of CD3 and/or CD28 via bead-coupled monoclonal antibodies led to poorer cell recovery as compared to cultures in the presence of recombinant human interleukin 2 alone (Figure 2C). Bypassing membrane-proximal signaling using PMA and ionomycin also reduced the frequency of viable cells, in this case from 76% to 27% (Figure 2D). These data suggest that the downstream signaling machinery of the TCR complex and CD28 is principally intact in T8-28 cells and that the observed induction of cell death upon stimulation of these pathways may be harnessed for therapeutic purposes. Pre-cautions [7], [8], however, need to be taken when targeting either the TCR complex or CD28 with monoclonal antibodies in humans as a profound activation of normal T cells may induce a toxic ‘cytokine storm’ [9], [10].

Therapeutically, allogeneic bone marrow transplantations may, of course, also be an option for the T8-28 lymphoma as, indeed, for human PTCL [11]. Most likely, a key component of any graft versus tumor effect *in vivo* is the recognition and killing of malignant cells by allo-reactive cytotoxic T cells [12]. As shown in Figure 2E, T8-28 cells were susceptible to killing by allogeneic CD8^+^ T cells *in vitro* suggesting that a graft versus tumor effect may also be elicited upon allogeneic bone marrow transplantation *in vivo*.

Taken together, the T8-28 cells constitute a transplantable PTCL line with, at least, partially defective effector functions but maintained susceptibility to killing by allogeneic T cells *in vitro*. This suggests that this newly established model could provide a basis for the development of novel immunotherapeutic approaches for PTCL in humans.

## Materials and Methods

### Ethics Statement

All experiments were performed according to the German regulations for animal experimentation and approved by the Regierung von Unterfranken as the responsible authority (Permit Number 55.2–2531.01-82/08 and -28/10).

### Mice

BALB/c.OlaHsd and C57BL/6.OlaHsd mice were obtained from Harlan-Winkelmann and were used for experiments between six and twelve weeks of age. BALB/c mice were irradiated at the age of nine to ten weeks.

### Adoptive transfers of T8-28 cells *in vivo*

1×10^7^, 5×10^6^ or 3×10^3^ freshly thawed T8-28 cells were adoptively transplanted intravenously into either un-manipulated or irradiated syngeneic BALB/c.OlaHsd mice as described [13]. After adoptive transfer of T8-28 cells mice were monitored for clinical signs of terminal lymphoma such as severe hunching, poor grooming, lack of spontaneous activity, severe respiratory distress and/or splenomegaly and animals with terminal disease were killed for humane reasons.

### Fluorescence-activated cell sorting

The following monoclonal antibodies were used (all Becton Dickinson or Biolegend unless stated otherwise): anti-CD3-FITC (fluorescein isothiocyanate), anti-CD4 Alexa Fluor® 647, anti-CD8α PE-Cy5 (phycoerythrin-Cy5), anti-CD8β PE, anti-CD25 FITC, anti-T cell receptor (TCR)β PE, anti-TCR Vβ 2, 3, 4, 5.1&5.2, 6, 7, 8.1&8.2, 8.3, 9, 10b, 11, 12, 13, 14 and 17a FITC (BD staining panel), anti-Ki-67 PE, anti-Granzyme B PE (eBioscience) and unconjugated anti-CD28 (clone E18) [14], [15]. Gαmouse Ig PE (Dianova) was used to detect bound anti-CD28 mAb.

Stainings, including intracellular stainings, were performed with up to 2×10^5^ T8-28 as described [13].

### Cytospins

Freshly thawed T8-28 cells and purified (99%) lymph node T cells from a C57BL/6 mouse, obtained by negative magnetic selection of cells expressing CD11b, CD11c, CD19, B220, CD49b, CD105, MHC-class II and Ter-119 according to the manufacturer's instructions (Miltenyi), were spun onto slides and stained using the Diff-Quik® staining kit (Medion Diagnostics).

### Analysis of the T cell receptor β chain by polymerase chain reaction DNA sequencing

RNA of T8-58 cells was extracted by guanidinium thiocyanate-phenol-chloroform extraction (TriZol) and transcribed into cDNA using first strand synthesis kit (Fermentas). PCR of cDNA was performed using Vβ2-leader (5′-GTGGCAGTTTTGCATTCTGTGCCT-3′) and Cbeta specific (5′-ACAGTCTGCTCGGCCCCAGG-3′) primers and Phusion polymerase (Finnzymes). The PCR product was separated by agarose gel electrophoresis. The most prominent band of about 800 bp was extracted and sequenced on an ABIMed 3100 machine. The sequence was analyzed by IMGT/V-QUEST programme version: 3.2.20 [16].

### *In vitro* cultures

All in vitro cultures were carried out using RPMI 1640 medium (PAA) supplemented with 10% heat-inactivated fetal calf serum, 1 mM sodium pyruvate, non-essential amino acids, 100 U/ml penicillin and 100 µg/ml streptomycin, 30 μM mercaptoethanol and 2 mM L-glutamine (all Gibco). All cells were incubated at 37°C in the presence of 5% CO_2_.

#### Cytotoxic activity of T8-28 cells

To assess the cytotoxic potential of T8-28 cells, a fixed number of CFSE-labeled (carboxyfluorescein succinimidyl ester diacetate; 5 µM; invitrogen) A20J cells were co-incubated with freshly thawed T8-28 cells in a 96 well V bottom plate at different effector to target cell ratios for four hours. To trigger re-directed lysis either anti-CD3 mAb 145-2C11 (Biolegend) or anti-β T cell receptor mAb H57-H7 (BD) were added at a final concentration of 10 µg/ml. To assess the susceptibility of A20J cells towards apoptotic stimuli, they were incubated with the anti-CD95 mAb Jo2 (BD) at a final concentration of 0.1 µg/ml. As a positive control for T cell-mediated cytolysis we used ‘Con A blasts’, i.e. lymph node cells (1×10^6^/ml) of a BALB/c.OlaHsd mouse which we had pre-activated with Concanavalin A (Con A; 5 µg/ml; Sigma) for three days *in vitro*. After the killing period, viability of the A20J target cells was determined by first counter-staining the cells with prodidium iodide and AnnexinV APC (BD) followed by fluorescence-activated cell sorting analysis.

#### Susceptibility of T8-28 cells to killing by allogeneic T cells

To generate H-2^d^-specific cytotoxic T cells, pooled spleen and lymph node cells (1×10^6^/ml) of C57BL/6 mice (H-2^b^) were cultured in the presence of irradiated BALB/c splenocytes (7.5×10^5^/ml, H-2^d^) and 10^-7^ M recombinant human interleukin 2 (Proleukin®, Novartis) for six days. In parallel cultures C57BL/6 spleen/lymph node cells depleted of CD8^+^ cells by negative magnetic separation were used. To deplete CD8^+^ cells (99%), the spleen/lymph node cell suspension was first stained with an anti-CD8α FITC mAb (BD) followed by anti-FITC beads (Miltenyi) and passage over an LD column according to the manufacturer's instructions (Miltenyi). In further parallel cultures, 2×10^6^ C57BL/6 total spleen/lymph node cells were cultured in the presence of recombinant human interleukin 2 only, i.e. without BALB/c splenocytes. After the pre-incubation period the effector cells were washed and co-incubated with a fixed number of 1×10^4^ CFSE-labeled T8-28 in a 96 well V bottom plate for four hours. Viability of the T8-28 target cells was, again, determined by first counter-staining the cells with propidium iodide and AnnexinV APC (BD) followed by fluorescence-activated cell sorting analysis.

#### Short term cultures

For short-term activation, freshly thawed T8-28 cells (1×10^6^/ml) were re-stimulated in the presence of phorbol myristate acetate (PMA)/ionomycin (5/500 ng/ml, Sigma) for four h before fluorescence-activated cell sorting analysis.

#### Cytokine- and mAb-mediated stimulations

To study the impact of recombinant human IL-2 (Proleukin®, Novartis), recombinant mouse interleukin 7 (R&D) and recombinant mouse interleukin 15 (Biolegend) on the growth and survival of T8-28 cells *in vitro*, 1×10^6^ T8-28 cells/ml (48 well plate, Greiner) were cultured in the presence of 10^−6^ M of exogenously added cytokine. After re-suspension, 200 µl were removed from each well on days one, two and five after initiation of the cultures.

The impact of CD3 and/or CD28 stimulation on T8-28 cell proliferation and survival were studied by incubating freshly thawed T8-28 cells (1×10^6^/ml) with 2.5×10^6^ Dynabeads® Pan Mouse IgG (Dynal) coated with either 10 µg/ml anti-CD3 mAb 145-2C11 (Biolegend), 10 µg/ml anti-CD28 mAb E18 [14], [15], 10 µg/ml mIgG_1_ mAb PPV-06 (Exbio) or 1 µg/ml anti-CD3 plus 0.5 µg/ml anti-CD28 mAb in the presence of 10^−6^ M recombinant human interleukin 2. In parallel cultures, only recombinant human interleukin 2 was added.

To determine absolute numbers of viable T8-28 cells at each time point, either 2×10^5^ of the freshly thawed T8-28 cells or all cells contained in 200 µl of suspension of the cultured cells (initially also 2×10^5^ cells) were stained with propidium iodide and AnnexinV APC before 1×10^4^ unlabeled CaliBRITE™ beads (BD) were added. After fluorescence-activated cell sorting analysis the absolute number of viable cells was determined as follows: (1×10^4^/number of beads recorded) x number of AnnexinV^-^ propidium iodide^-^ T8-28 cells recorded.

### Statistics

Summary graphs were generated using Excel^©^ 12.3.0 (Microsoft) and p-values are the results of two-way ANOVA testing (GraphPad Prism 4.0c^©^). P<0.05 was considered statistically significant.

## Supporting Information

Table S1 In addition to the data shown in Figure 1B, D and E, further fluorescence-activated cell sorting analyses of T8-28 cells rendered the results summarized in this table.
(PDF)
