# Supplementary material for: Characterization of a New Mouse Model for Peripheral T Cell Lymphoma in Humans
Source: PLoS One. 2011 Dec 5;6(12):e28546. doi: 10.1371/journal.pone.0028546 (PMC3230627; doi:10.1371/journal.pone.0028546)
Supplement: Table S1 — In addition to the data shown in Figure 1B, D and E, further fluorescence-activated cell sorting analyses of T8-28 cells rendered the results summarized in this table. (PDF) [file pone.0028546.s001.pdf]

Table S1

Marker	Expression	mAb	Manufacturer
B220	negative	RA3-6B2	BD
CD44	low	IM7	BD
CD49b	negative	Dx5	BD
CD62L	low	MEL-14	BD
CD69	negative	H1.2F3	BD
CD122	positive	5H4	BD
CD127	low/-	SB/199	Biolegend
CD132	positive	TUGm2	Biolegend
CD152	positive	UC10-4F10-11	BD
D^d	positive	34-2-12	BD
Foxp3	negative	FJK-16s	eBioscience
I-A^d	negative	AMS-32.1	BD
Killer lectin-like receptor G1	negative	2F1	BD
